# Knowledge and attitudes about stroke among Egyptian adults: results from an online cross-sectional survey

**DOI:** 10.1186/s12889-026-27083-z

**Published:** 2026-04-13

**Authors:** Ahmed Amir Samir, Ahmed Hussein Elamir, Ahmed W. Hageen, Mohamed Yasser El-mezayen, Ibrahim Ali Kabbash, the Stroke Collaborative Group, Abdurrahman Smeary Mohamed, Safaa Hassan Zaki, Tiffany John Awadalla, Rodina Mohamed Elsayed, Wael Reda Attalla, Manar Khaled, Mohamed Ashraf Hussein, Ahmed Attef, Doaa Ali Kandil, Mohamed Samar Shalaby, Alshimaa Hesham Draz, Ahmed Soliman Ibrahim, Ahmed Elssaman, Passant Saeed Ahmed, Reem Salah Algahory, Ahmed Hatem Ibrahim, Aya Ali Abdelrady, Amr Mohamed Shawkat, Mostafa Mohamed Elsayed, Adel Marwan Mohamed, Mohamed Emad Abdelmonem, Fatma Fathy Bahnasy, Esraa Amer Sola, Shaimaa Abdelhamid Altoury, Michael Saad Ramzy, Ibtisam Mohamed Mouslawi, Amal Farghaly, Eman Soliman, Mostafa Foly Shaban, Mohamed Fawzi Hemida, Bishoy Gebraiel Malak, Ahmed Mosaad Sobhi, Engy Elsayed Hassan, Rana Ashraf Mohamed, Basma Mohamed Ghoneim, Mariam El-Saeed

**Affiliations:** 1 Faculty of Medicine, Al-Azhar University, Cairo, Egypt; 2 Faculty of Medicine, Mansoura University, Dakahlia, Egypt; 3 Faculty of Medicine, Tanta University, Tanta, Egypt; 4 Faculty of Medicine, Alexandria University, Alexandria, Egypt; 5 Public Health & Community Medicine—Faculty of Medicine, Tanta University, Tanta, Egypt; 6 Department of Public Health and Community Medicine, Faculty of Medicine, Tanta University, Tanta, Egypt

**Keywords:** Stroke, Knowledge, Awareness, Attitude, Acute stroke response, Egypt, cross-sectional study

## Abstract

**Background:**

Globally, stroke is a major cause of morbidity and mortality. Assessing the public understanding of stroke is crucial, as it can improve stroke services and minimize the time between the onset of symptoms and the delivery of thrombolysis and thrombectomy, thereby improving patient outcomes and reducing mortality. This study aimed to assess stroke knowledge among Egyptian adults and identify factors influencing their knowledge.

**Methods:**

A cross-sectional study was conducted using an anonymous self-administered Arabic questionnaire distributed via various social media platforms. The survey assessed knowledge about stroke risk factors, symptoms, consequences, and appropriate responses to stroke symptoms.

**Results:**

A total of 4516 participants completed the questionnaire, with 64.3% being females. Most participants were aged 18–29 (82.6%), and those working or studying in the medical field represented 52.9%. Knowledge of stroke risk factors, symptoms, and consequences was prevalent among participants, with 65.9% having a good level of knowledge. Internet and social media platforms were the most common sources of information (56.6%). Having a high level of education, medical field of work, reporting having higher income level, having heard about stroke, a positive family history of stroke, and knowing someone with stroke were all predictors of a good level of stroke knowledge.

**Conclusion:**

The study revealed a high level of knowledge among study participants. However, additional efforts are necessary to promote awareness through targeted educational interventions and campaigns, especially among vulnerable populations.

## Introduction

Stroke represents a substantial global health concern. One in four individuals faces the risk of experiencing a stroke in their lifetime [1]. In 2021, stroke ranked as the third most prevalent cause of death, accounting for 7.3 million deaths annually. Moreover, 160.5 million global Disability-Adjusted Life Years (DALYs) were attributed to stroke, which stands as the fourth leading cause of disability, constituting 5.6% of the total DALYs. Furthermore, over 87.2% of mortality and more than 89.4% of stroke-related DALYs occur in low- and middle-income countries [2]. Various risk factors are highly associated with stroke, including obesity, dyslipidaemia, insufficient exercise, smoking, heart problems, high blood pressure, diabetes mellitus, and alcohol consumption [3]. Nonetheless, lifestyle modifications and appropriate risk factor management can prevent a significant portion of strokes [4]. Stroke leads to cognitive decline, speech difficulty, emotional distress, and paralysis [5].

The American heart association/American Stroke Association guidelines recommend intravenous thrombolysis with recombinant tissue plasminogen activator (rt-PA) within 4.5 h of symptom onset as the standard care for acute ischemic stroke. It is effective and safe, decreasing mortality and long-term disability. However, the effectiveness of thrombolytic therapy is dependent on the time and extent of drug administration post-symptom onset [6]. Pre-hospital delay is one of the key factors that limits the percentage of acute stroke patients who receive recanalization therapy, which is affected by objective factors like age, sex, and regional economic level, and also by subjective factors like stroke awareness [7]. Numerous studies have demonstrated that delays in seeking and receiving treatment during the early stages of acute stroke remain a widespread issue, significantly affecting patient outcomes [8–10]. Similarly, in Egypt, pre-hospital delays remain a major issue. Zakaria et al. found that the most significant causes of extended onset to door are lack of patient or relative awareness of symptoms of stroke, waiting for symptoms to resolve spontaneously, and inadequate knowledge about the availability of immediate intervention for stroke [11]. A registry-based study from an Egyptian stroke centre demonstrated that many patients arrive beyond the therapeutic window, with 20.6% having received thrombolytic therapy [12]. 

Furthermore, the public’s poor knowledge regarding stroke symptoms and prevention practices is considered the main cause of this pre-hospital delay [13, 14]. Previous studies reported that public knowledge of stroke and appropriate action were substantially linked with greater knowledge of stroke-related symptoms and warning signs [15, 16]. Individuals who were aware of the early symptoms of stroke and risk factors were more likely to respond to these symptoms by seeking immediate health care [17]. In contrast, people with limited knowledge are less likely to recognize the early symptoms of stroke, which leads to delayed treatment and worsened outcomes [18]. Therefore, there is an urgent necessity to enhance the early recognition of stroke-related signs and symptoms and to mitigate delays in obtaining treatment. This may occur through increased public awareness regarding the different aspects of stroke, especially warning signs, which facilitates early detection and improved response rates.

Prevention of secondary stroke is challenging, especially in low- and middle-income countries, perhaps due to a lack of medication, low knowledge, poor medication compliance, and elevated out-of-pocket therapy expenses [19]. In Egypt, the incidence of stroke is 240/100,000, which translates into 150,000 to 250,000 new cases annually. Of them, 10% die within one month after a stroke. The rest often endure varying levels of disability, which contributes to a major healthcare challenge [20]. Furthermore, it is essential to note that thrombolysis and thrombectomy rates remain relatively low [21]. Studies conducted in the Gharbia, Cairo, Assiut, and Beni-Suef governorates of Egypt indicate that there is generally low public knowledge about stroke, specifically among rural Egyptian citizens. This disparity may be due to social and economic status and educational level [22, 23]. 

In Egypt, stroke services changed dramatically in 2014, when the Ain Shams University Specialized Hospital (ASU) stroke team began investigating the barriers to adequate acute stroke management in the prehospital and in-hospital settings. As a result, an action plan was established by the ASU team, including training for residents on reperfusion. Furthermore, other Egyptian universities began following the same path as ASU. The ASU stroke program demonstrated a growing rate of acute stroke treatment, including thrombolysis and thrombectomy [24]. Their efforts require raising public and primary care physicians awareness about stroke symptoms and the need for prompt treatment. While knowledge about stroke is a key component of stroke management and prevention, no comprehensive study has specifically addressed stroke knowledge, including symptoms, risk factors, and consequences, among the public in Egypt. Given the major changes in stroke services in Egypt, ongoing assessment of public knowledge is crucial to ensure effective utilization. Therefore, this study aims to assess the extent of stroke knowledge among Egyptians and determine the influencing factors that affect this knowledge. Additionally, our study aims to reach a wide population, emphasizing the impact of education and social media in shaping awareness. These tools can help target communities and promote stroke preparedness.

## Methods

### Study design, settings, and participants

This cross-sectional study was conducted between September and November 2024 using an anonymous, Arabic, self-administered online questionnaire distributed to the general population. Eligibility criteria included Egyptian citizens from all governorates or regions, aged 18 years or older, of both genders, and able to complete the questionnaire in Arabic. Non-Egyptians were excluded.

### Sample size calculation

The eligible study participants were recruited using the snowball and convenience sampling methods. The sample size was calculated using Epi Info statistical software version 7.2.6, with the following criteria: a 50% expected level of good awareness of stroke among the target population, a 95% confidence level, a 5% allowable margin of error, and a design effects factor of two to account for the non-random sampling. The sample size was 768 individuals from each region. The total recruited sample size was 4516 participants, which increased the study’s power and precision.

### Questionnaire

The Arabic questionnaire used in this study was adapted from a previously published survey developed and validated by Barakat et al. in the Arabic language [25, 26]. It aimed to assess stroke awareness and general knowledge among the general population and covered multiple aspects of stroke knowledge, including risk factors, symptoms, and consequences. The approximate duration required for answering the questionnaire was 10–15 min. The survey comprised four sections. The first section covered socio-demographic characteristics, including age, gender, marital status, field of study or work, residence area, educational level, and self-reported income. The general knowledge of stroke was assessed in the second section. Participants answered these questions: Stroke is a disease that (1) affects the brain, (2) is contagious, (3) is a hereditary disease, (4) is preventable, and (5) is an old person disease. Additionally, this section included questions about familiarity with stroke (having heard about stroke, family history of stroke, knowing someone with stroke, and personal experience with stroke). The third section evaluated knowledge about risk factors for stroke, including smoking, physical inactivity, old age, alcohol consumption, heart disease, diabetes, psychosocial stress, and hypertension. In addition, it assessed knowledge about stroke symptoms including: (1) sudden confusion or loss of consciousness; (2) sudden difficulty speaking; (3) sudden severe headache; (4) sudden numbness or weakness of the face, arms, or limbs, particularly on one side of the body; (5) sudden onset of memory loss; (6) sudden dizziness or loss of coordination; and (7) sudden blindness or double vision in one or both eyes. Finally, the fourth section covered the potential consequences of stroke, such as visual problems, movement problems, emotional and personality changes, memory and cognitive problems, and long-term disabilities. Additionally, three questions evaluated the participants’ reactions and attitudes towards a patient experiencing a stroke. Participants were given one point for every correct answer in the general knowledge, risk factors, symptoms, and consequences sections. The total score of knowledge ranged from 0 to 26. The total score of < 50% was considered as poor, 50–75% as fair, and > 75% as good. The Cronbach’s alpha was calculated for the questionnaire and was found to be 0.783, indicating good reliability.

### Data collection

An online link to an internet-based survey created with the “Google Forms” platform was distributed to the public on various social media platforms and community groups. To encourage participation from all governorates of Egypt, we divided the country into regions that include Greater Cairo, Alexandria, the Suez Canal, the Nile Delta, and Upper Egypt. We assigned 6–8 collaborators (the stroke collaborative group) to supervise data collection in each region and encourage participation to achieve the target sample. Before data collection, we trained them on survey dissemination techniques and data gathering protocols. The data was anonymously recorded by the participants, and no contact or personal information was recorded. The opening section of the questionnaire included an overview of the study and provided electronic informed consent with choices to complete or cancel the questionnaire. To avoid duplicate responses, the “collect IP addresses” feature was activated in Google Forms.

### Ethics approval and consent to participate

Participation in this study was voluntary, and it followed the principles of the Declaration of Helsinki in its entirety [27]. Everyone provided their informed consent electronically before participation. The study was ethically approved by the Institutional Review Board (IRB) of the Faculty of Medicine, Tanta University, Tanta, Egypt (Approval number: 36264PR850/9/24).

### Statistical analysis

The collected data was organized, tabulated, and statistically analyzed using SPSS version 26 (Statistical Package for Social Sciences), created by IBM in Chicago, Illinois, USA. For numerical variables, the range, mean, and standard deviation were calculated. The differences between the two mean values were assessed using Student’s t-test. For the categorical variables, the number and percentage were calculated, and differences between subcategories were tested by the chi-square test. Binary logistic regression was applied to identify independent predictors of having a good level of stroke knowledge (*≥* 75% of the total score). The dependent variable was stroke knowledge, which was dichotomized (good/poor), and the independent variables included in the logistic regression model were selected based on bivariate analysis (*p* < 0.05) and theoretical relevance. The Hosmer–Lemeshow goodness-of-fit test and Nagelkerke R² of 0.218 were included. Statistical significance was set at *p* < 0.05.

## Results

A total of 4516 participants completed the survey. The majority (82.6%) were aged 18–29, 64.3% were females, and 73.4% reported urban residence. Most participants had a university degree or higher (91.5%), and 52.9% were studying or working in the medical field. A moderate monthly income was reported by 75.4% of participants (Table 1).

**Table 1:** Demographic characteristics of the participants

Variables	Number (*n* = 4516)	%
Age in years:		
18–29	3733	82.6
30–50	603	13.4
> 50	180	4.0
Gender:		
Male	1614	35.7
Female	2902	64.3
Residence:		
Urban	3315	73.4
Rural	1201	26.6
Marital status:		
Single	3587	79.4
Married	872	19.3
Widow	21	0.5
Divorced	36	0.8
Highest educational degree:		
University or above	4133	91.5
Secondary	266	5.9
Primary	117	2.6
Field of study or work		
Non-medical	2127	47.1
Medical	2389	52.9
Self-reported income:		
Low	805	17.8
Moderate	3403	75.4
High	308	6.8

**Fig. 1:**
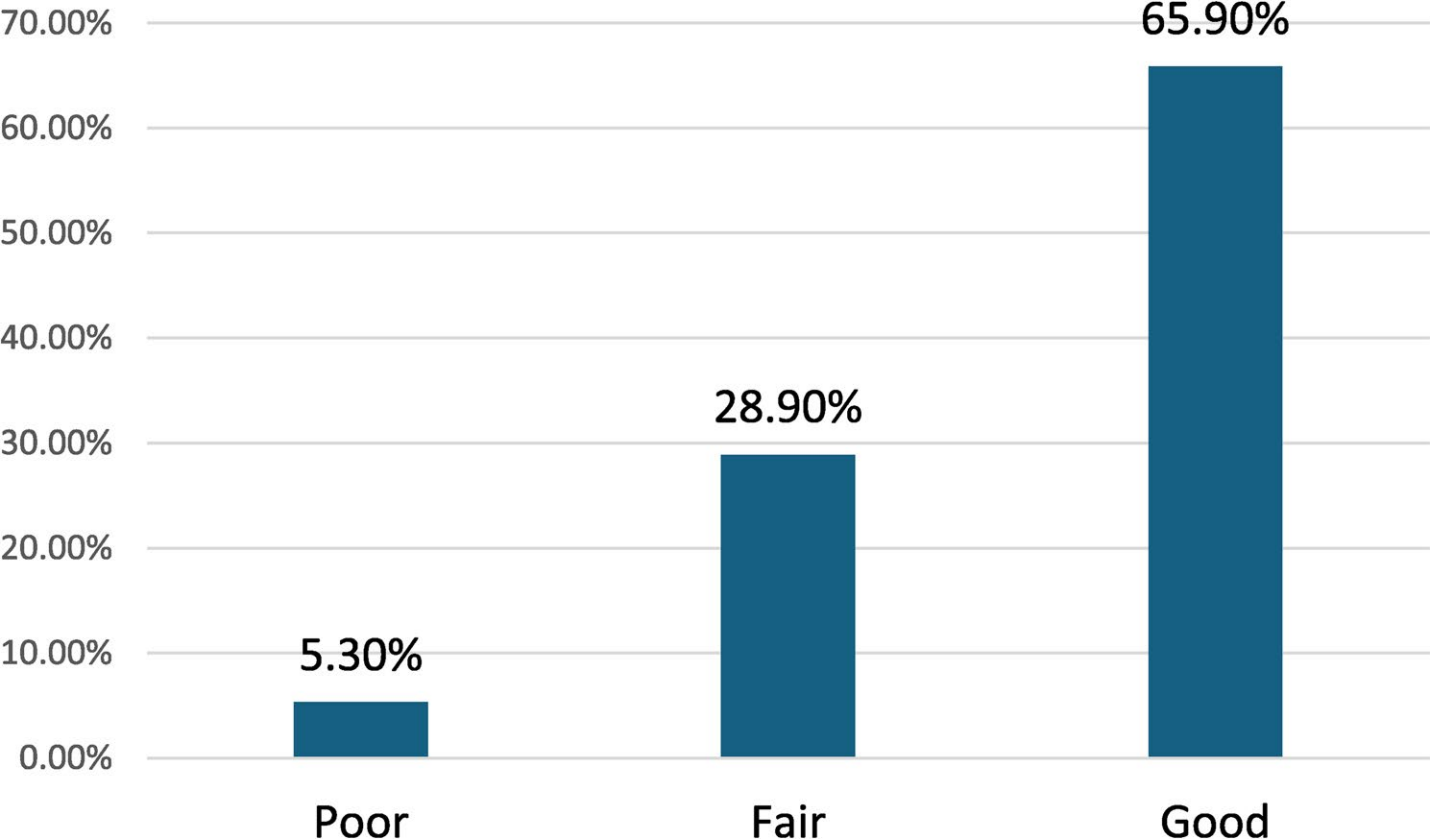
Distribution of participants by their level of stroke knowledge

**Fig. 2:**
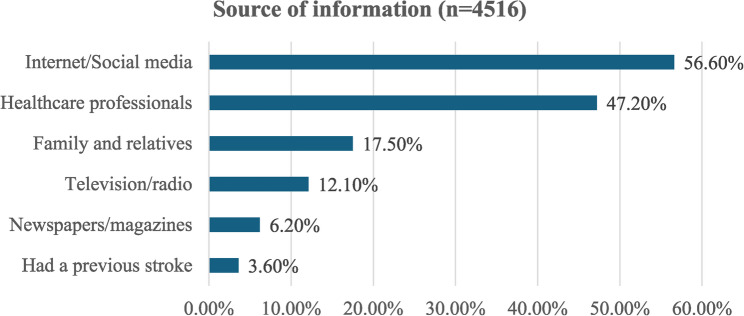
Sources of information about stroke among participants (*n* = 4516)

Those with a good level of knowledge represented 65.9%, while 28.9% had a fair level of knowledge (Fig. 1). The most common sources of stroke information were the internet/social media, healthcare professionals, and family/relatives (56.6%, 47.2%, and 17.5%, respectively) (Fig. 2).

A significantly higher percentage of females had heard about stroke (89.5%) compared to males (87.2%) (*p* = 0.017). Overall, 43.5% of participants reported knowing someone who had experienced a stroke, 13% reported a family history of stroke, and 1.2% had experienced a stroke themselves. (Table 2)

**Table 2:** Gender Differences in familiarity with stroke and reported stroke symptoms, risk factors, and consequence

Variables	Male (*n* = 1416)		Female (*n* = 2902)		Total (*n* = 4516)		X^2^	*P*
	*n*	%	*n*	%	*n*	%		
Familiarity with stroke								
Having heard about stroke	1407	87.2	2598	89.5	4004	88.7	5.707	0.017*
Family history of stroke	206	12.8	379	13.1	585	13.0	0.083	0.773
Knowing someone with stroke	690	42.8	1276	44.0	1966	43.5	0.627	0.429
Personal experience with stroke	19	1.2	35	1.2	54	1.2	0.007	0.932
Stoke general knowledge:								
Stroke primarily affects brain	1500	92.9	2729	94.0	4229	93.6	2.116	0.146
Stroke is contagious	18	1.1	26	0.9	44	1.0	0.517	0.472
Stroke affects old persons	86	5.3	146	5.0	232	5.1	0.188	0.664
Stroke is hereditary	261	16.2	466	16.1	727	16.1	0.010	0.921
Stroke can be prevented	1455	90.1	2625	90.5	4080	90.3	0.111	0.738
Stroke Symptoms								
Sudden dizziness or loss of coordination	1222	75.7	2283	78.7	3505	77.7	5.221	0.022*
Sudden blindness or double vision	1243	77.1	2314	79.7	3557	78.8	4.444	0.035*
Suddenly severe headache	1140	70.6	2107	72.6	3247	71.9	1.999	0.157
Sudden onset of memory loss	632	39.2	1089	37.5	1721	38.1	1.171	0.279
Loss of consciousness	1195	74.0	2184	75.3	3379	74.8	0.808	0.366
Sudden numbness or weakness	1206	74.7	2275	78.4	3481	77.1	7.921	0.005*
Sudden difficulty speaking	1294	80.2	2361	81.4	3655	80.9	0.943	0.332
Stroke Consequences								
Long term disabilities	1272	78.8	2339	80.6	3611	80.0	2.072	0.150
Movement / functional problems	1389	86.1	2567	88.5	3956	87.6	5.485	0.019*
Cognitive problems	1283	79.5	2382	82.1	3665	81.2	4.547	0.033*
Visual problems	1093	67.7	2049	70.6	3142	69.6	4.082	0.043*
Personality changes	978	60.6	1773	61.1	2751	60.9	0.109	0.741
Stroke Risk Factors								
Hypertension	1469	91.0	2693	92.8	4162	92.9	4.559	0.033*
Smoking	1289	79.9	2342	80.7	3631	80.4	0.464	0.496
Diabetes mellitus	987	61.2	1853	63.9	2840	62.9	3.240	0.072
High cholesterol	1298	80.4	2383	82.1	3681	81.5	1.976	0.160
Aging	1341	83.1	2374	81.8	3715	82.3	1.164	0.281
Heart Disease	1257	77.9	2380	82.0	3637	80.5	11.293	0.001*
Excessive alcohol	1314	81.4	1256	81.2	3670	81.3	0.035	0.851
Psychosocial stress	1461	90.5	2647	91.2	4108	91.0	0.605	0.437
Physical inactivity	1180	73.1	2160	74.4	3340	74.0	0.940	0.332

*p value < 0.05

Of participants, 93.6% recognized that stroke primarily affects the brain, and 90.3% believed it can be prevented. Concerning stroke symptoms, significantly more females reported sudden dizziness or loss of coordination and sudden blindness or double vision (78.7% and 79.7%, respectively) as compared to males (75.7% and 77.1%, respectively) (*p* = 0.002 and 0.035, respectively). Other symptoms were known by more than 70% of participants, except for sudden memory loss, which was reported by 38.1%. Most participants reported the consequences of stroke. Movement or functional problems, cognitive problems, and visual problems were significantly more recognized by females (88.5%, 82.1%, and 70.6%, respectively) compared to males (86.1%, 79.5%, and 67.7%, respectively) (*p* = 0.019, 0.033, and 0.043, respectively). The majority identified the different risk factors for stroke. Hypertension was reported by 92.8% of females, which was significantly higher than the 91% reported by males (*p* = 0.033). Heart disease was reported by 82% of females and 77.9% of males, a statistically significant difference (*p* = 0.001). (Table 2)

**Fig. 3:**
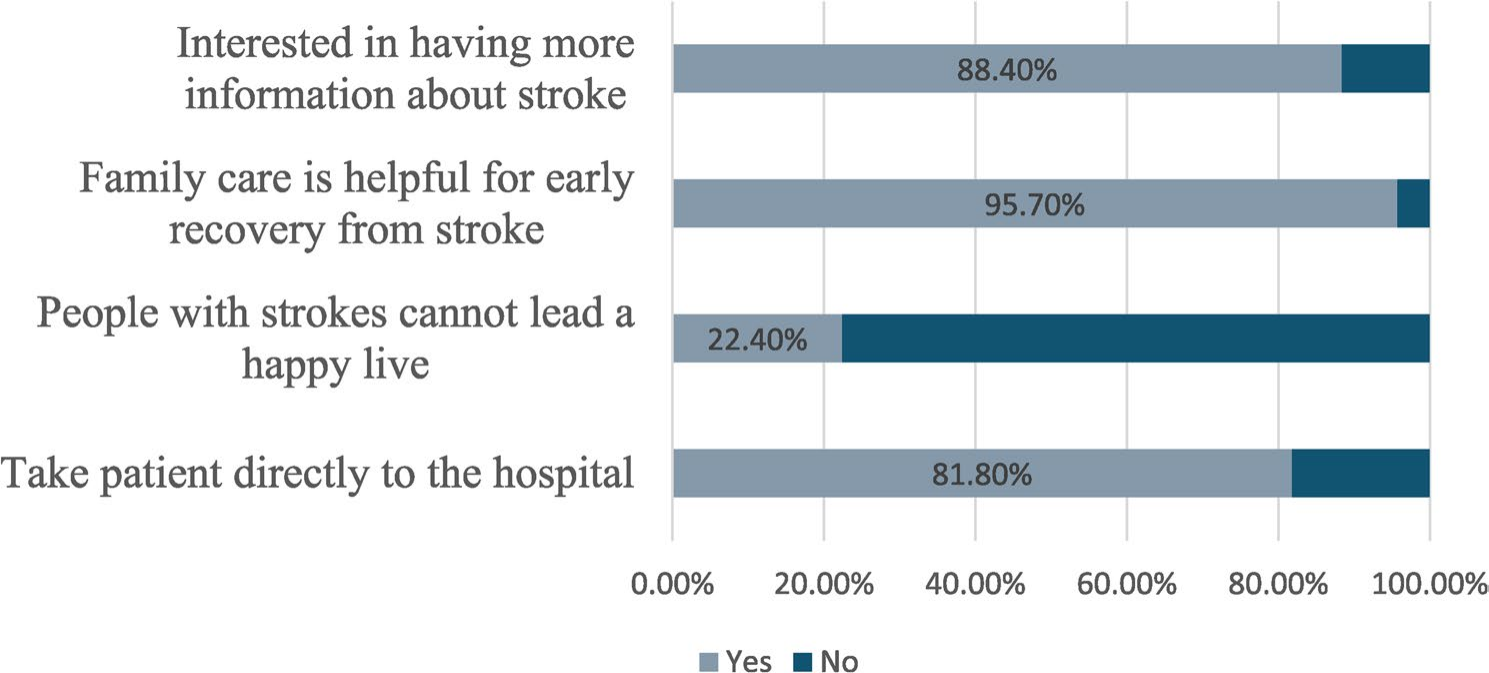
Attitude and reaction towards stroke (*n* = 4516)

Regarding the attitudes of the participants towards stroke, 95.7% believed that family care is helpful for early recovery, 22.4% believed that people who have had a stroke can’t live a happy life, and 81.8% reported that they would take a person to the hospital as soon as stroke symptoms appeared. Among participants, 88.4% reported curiosity about more information about stroke. (Fig. 3)

The younger age group (18–29) and females had significantly higher knowledge scores than the older age group and males (*p* = 0.004 and 0.011, respectively). Those with university education were found to have a mean score of 20.88 ± 4.42, which was significantly higher than 18.33 ± 4.86 among those with a lower educational level (*p* < 0.001). Participants with lower levels of income had a significantly lower mean knowledge score than those with moderate levels (20.13 + 4.59 and 20.45 + 4.91, respectively; *p* < 0.001). Studying or working in the medical field, having heard about stroke, a positive family history of stroke, and knowing someone with stroke were associated with higher mean scores of stroke knowledge (*p* < 0.001). Participants without a history of stroke scored significantly higher in total knowledge than those who had experienced a stroke (*p* < 0.001). (Table 3)

**Table 3:** Factors affecting total score of stroke knowledge among studied participants

Variables	Total knowledge score		t	*p*
	Range	Mean *±* SD		
Age in years			2.849	0.004*
18–29	2–26	20.75 *±* 4.47		
* ≥* 30	3–26	20.23 *±* 4.70		
Gender			2.535	0.011*
Males	2–26	20.43 *±* 4.63		
Females	2–26	20.79 *±* 4.45		
Residence			0.234	0.815
Urban	2–26	20.65 *±* 4.56		
Rural	3–26	20.69 *±* 4.40		
Educational level:			9.910	< 0.001*
Below university	2–26	18.33 *±* 4.86		
University or above	2–26	20.88 *±* 4.42		
Field of study or work			27.748	< 0.001*
Non-medical	2–26	18.82 *±* 4.58		
Medical	2–26	22.30 *±* 6.77		
Self-reported income:			7.713#	< 0.001*
Low	3–26	20.13 *±* 4.59		
Moderate	2–26	20.81 *±* 4.46		
High	2–26	20.45 *±* 4.91		
Having heard about stroke			14.992	< 0.001*
No	2–26	17.55 *±* 5.06		
Yes	2–26	21.06 *±* 4.29		
Family history of stroke			7.978	< 0.001*
No	2–26	20.45 *±* 4.58		
Yes	2–26	21.89 *±* 3.88		
Personal experience with stroke			5.102	< 0.001*
No	2–26	20.71 *±* 4.48		
Yes	2–26	16.74 *±* 5.69		
Knowing someone with stroke			14.885	< 0.001*
No	2–26	19.25 *±* 4.79		
Yes	2–26	21.75 *±* 3.88		

*p value < 0.05

#Bonferroni test: low category significantly different from moderate

The multivariate logistic regression analysis showed that participants with higher education levels (University or higher), moderate to high income, having heard about stroke, have family history of stroke, and knowing someone with stroke were significantly more likely to have a good level of stroke knowledge [ Exp(B) = 1.739, 1.223, 2.853, 1.582 and 1.786, respectively]. Those with non-medical field of study were less likely to have good level of knowledge (Exp (B) = 0.251). Age, sex and residence were not found as independent predictors for knowledge level. (Table 4)

**Table 4:** Binary logistic regression model for the predictors affecting good level stroke knowledge among studied participants

Variables	Good level knowledge *(>* 75% of total score)				
	B	Wald	*p*-value	Exp (B)	95% CI
Age in years (> 30)	0.098	1.099	0.294	1.101	0.920–1.317
Sex (female)	0.007	0.009	0.923	1.007	0.875–1.159
Residence (rural)	0.057	0.524	0.469	1.058	0.908–1.233
Educational level: *≥*University	0.553	22.100	< 0.001*	1.739	1.381–2.189
Field of study or work (Nonmedical)	-1.381	355.073	< 0.001*	0.251	0.218–0.290
Income: (Moderate to high)	0.201	5.079	0.024*	1.223	1.027–1.457
Having heard about stroke	1.049	108.833	< 0.001*	2.853	2.343–3.475
Family history of stroke	0.459	13.948	< 0.001*	1.582	1.244–2.013
Personal experience with stroke	-1.397	21.244	< 0.001*	0.247	0.137–0.448
Knowing someone with stroke	0.580	60.760	< 0.001*	1.786	1.544–2.067

*p value < 0.05

Hosmer and Lemeshow test: Chi square = 16.966, *p* = 0.030

Nagelkerke R^2^ =0.218

## Discussion

Health literacy encompasses the knowledge of health and healthcare, the ability to access, process, and understand health information effectively, and the successful application of this knowledge for self-care and collaboration with medical professionals [28, 29]. The present study aimed to assess the level of stroke knowledge, identify knowledge gaps, and use these findings to guide interventions to enhance stroke knowledge and promote appropriate responses to stroke symptoms, helping the community achieve better health outcomes.

### Stroke knowledge among studied participants

In 2018, Farrag et al. showed low public stroke knowledge in four Egyptian governorates, with a median score of 35.7% [22]. Elhassanien et al. (2023) reported lower overall stroke knowledge and awareness, especially in rural and less educated groups, attributing this primarily to low socioeconomic status and lower educational levels [23]. Additionally, a recent Egyptian study utilizing an online survey demonstrated inadequate knowledge about stroke [30]. However, our results showed a relatively good level of stroke knowledge, with 65.9% of the sample demonstrating a good level of knowledge, as most participants recognized key risk factors, symptoms, and consequences. The observed level of stroke knowledge in this study likely reflects a more privileged subgroup rather than the broader Egyptian population, as our study sample predominantly consisted of young, urban, highly educated individuals, and a substantial proportion (over 50%) were currently studying or working in the medical field, which limits generalizability to the broader population. Therefore, comparisons with previous studies conducted in Egypt should be interpreted with caution. These observed differences are more reasonably explained by variations in recruitment methods (e.g., online surveys vs. general population sampling) rather than a genuine national improvement in knowledge. Hence, future research involving more diverse and representative populations is recommended.

### Knowledge of stroke risk factors, symptoms, and consequences

Regarding general stroke knowledge, our cohort demonstrated high levels of awareness. They revealed that stroke primarily affects the brain (93.6%) and that stroke is preventable (90.3%). Participants showed high recognition of common stroke symptoms, with awareness exceeding 70% for most symptoms. However, recognition of sudden memory loss as a stroke symptom was notably lower, indicating a potential gap in understanding cognitive manifestations of stroke. Moreover, our study shows that participants were most familiar with common stroke risk factors, including hypertension, psychosocial stress, heart disease, dyslipidaemia, diabetes, and smoking, reflecting knowledge of the main factors that increase the risk of stroke [31]. Furthermore, participants identified the serious consequences of stroke, including movement/functional problems, cognitive problems, and visual problems. These findings align with existing literature on stroke sequelae, which emphasizes the multifaceted nature of stroke-related disabilities [32].

Internationally, findings align with studies from other Middle Eastern countries, where higher stroke knowledge is often linked to being females, urban residents, and those with higher educational attainment [25, 26, 33–37]. Therefore, the high recognition of stroke aspects in our sample likely stems from the overrepresentation of educated, digitally connected participants, and those from the medical field, groups that typically exhibit superior health literacy [38, 39]. Such factors could indicate sampling bias rather than a true population-level improvement. These findings indicated the need for targeted public education to improve awareness of specific, clinically significant stroke symptoms to encourage timely recognition and response, reducing prehospital delay and improving outcomes. Targeted initiatives should concentrate on developing community-based stroke education and support networks, expanding media outreach beyond digital platforms, and closing knowledge gaps in underprivileged populations through customized educational programs.

### Attitudes towards stroke

Most participants were willing to seek immediate medical care if they witnessed someone experiencing stroke symptoms. This is a generally positive finding, as it can greatly enhance stroke outcomes by recognizing symptoms early and taking appropriate action. Similarly, most participants in other studies understood the significance of attending an emergency room at a hospital as soon as possible once a stroke is suspected [25, 26, 33]. In addition, 88.4% expressed interest in learning more about stroke. This is an initiative-taking outcome that can be leveraged in educational programs. Thus, appropriate educational interventions targeting specific knowledge gaps may encourage caregivers while enhancing their ability to provide efficient support to stroke survivors. However, the sample’s demographic suggests such interest may not extend to underserved groups, where barriers like low internet penetration persist. Furthermore, about 22.4% believed that stroke survivors are unable to lead fulfilling lives, representing a prevalent stigma associated with stroke rehabilitation. This underscores the importance of correcting these misconceptions and fostering a more inclusive and supportive environment for those recovering from stroke.

### Source of information about stroke

In the present study, the most common information sources for stroke were the internet and social media platforms, followed by healthcare professionals, consistent with trends observed in other studies [25, 35, 36]. This digital dominance contributes to our cohort characteristics, as online platforms facilitate access for young, educated users. However, the reliance on internet/social media platforms to acquire health-related information emphasizes the central role of digital platforms in disseminating validated health information. This highlights the crucial opportunity for public health initiatives to leverage digital platforms through tailored campaigns and strong collaboration between health authorities and these platforms to disseminate reliable health content, combat misinformation, and improve outreach. This is particularly relevant in the Middle East and North Africa region, where there is limited healthcare infrastructure, which increases reliance on digital sources for health-related information [40].

### Predictors of knowledge about stroke among the studied participants

Similar to previous studies, females exhibit greater stroke knowledge [25, 33–35, 37]. However, this association was not significant after controlling for confounding, suggesting that gender may not independently predict stroke knowledge. This could be explained by the fact that women are typically more proactive in accessing health information and services and spend more time seeking information than men [41, 42]. They are usually the main caregivers of the family and more likely to be the main support for ill family members. In contrast to our study, previous studies demonstrated that urban populations had better knowledge compared to rural populations, as well as more appropriate responses to acute stroke [22, 23]. This could be attributed to better accessibility to information resources and health services than in rural populations [43]. Furthermore, educational attainment was a strong predictor of stroke knowledge, with higher educational levels associated with a good level of stroke knowledge. This is consistent with a multinational local study among several Arab populations [33]. While our sampling method has its advantages, including the ease of recruitment through the online self-administered questionnaire, a method that tends to attract more educated and digitally literate individuals, it offers a unique chance for targeting this group for community awareness initiatives, enabling those individuals to help disseminate information to their families and communities.

The present study identified a potential impact of the personal experience of stroke on participants’ knowledge. Those who knew a stroke patient and had a family history of stroke were significantly more aware of stroke. Similarly, this finding was observed in a recent study among Egyptians [30]. This demonstrates that personal and familial experiences with stroke play a vital role in influencing awareness and understanding of the condition. This increased knowledge may result from frank conversations about health risks between families, which emphasize the value of family education in stroke prevention efforts.

Our results revealed that stroke-free individuals had the highest total score of knowledge when compared with stroke survivors. This low knowledge score among stroke sufferers may be attributed to post-stroke cognitive impairment, which can affect memory, attention, processing speed, and executive functions [44]. This can further undermine health literacy and impact on health-related information is understood, remembered, and applied [45–47]. A previous study also reported that patients who have experienced a stroke find it extremely difficult to find and interpret online health resources [48]. In the WHO European Region, higher socioeconomic position is linked to greater knowledge of stroke risk factors and symptoms [49]. Similarly, our study showed that moderate to high income levels are positively associated with a good level of stroke knowledge. These findings highlight the need for targeted public health initiatives and educational programs aimed at increasing stroke knowledge, especially among non-medical, lower socioeconomic, less educated, and rural groups.

#### Recommendations and action plan

Despite Egypt’s leading position in various stroke services compared to many African countries, awareness regarding stroke remains insufficient. This may hinder early detection, delay hospital admission, influence secondary prevention measures, post-discharge care, and follow-up compliance. Collaborative action is needed from policymakers, medical professionals, and non-government organizations to focus on enhancing public awareness through tailored campaigns such as wider use of popular social media platforms in Egypt, organized community events, school-based education to incorporate information concerning stroke risk factors and prevention into school health curriculum, radio campaigns in rural areas, or collaboration with local community leaders. Operational public health initiatives should encompass improving stroke care infrastructure and providing nationwide technology-facilitated awareness programs. Future qualitative research is needed to better explore the sociocultural and psychological factors influencing stroke response. Longitudinal studies are recommended to determine the impact of public health initiatives. Although our findings offer valuable insights into the studied population, the results may not fully represent the older population, people from rural areas, or less educated participants. With such disparities between these populations in attitudes toward stroke, access to care, and health awareness, the generalizability of our findings might be affected. Thus, future research is needed to explore these subgroups more thoroughly.

#### Study strengths and limitations

Our study has several notable strengths. The generalizability and representativeness of the findings are improved by the inclusion of a large sample size (*n* = 4516) from diverse regions throughout Egypt. Furthermore, the geographic scope, including both rural and urban areas, provides a significant understanding of stroke in different socioeconomic backgrounds. Moreover, this study used a voluntary, anonymous, well-structured survey to ensure a comprehensive evaluation of stroke awareness, including risk factors, early warning signs, and consequences. The study explored sociodemographic determinants, particularly the impact of education and social media on stroke knowledge. Despite these strengths, the study has some limitations. Using convenience and snowball sampling methods could result in selection bias and the exclusion of people with limited accessibility to internet sources or digital literacy. Another limitation of our study is the demographic characteristics of the sample, which were predominantly composed of younger individuals, urban residents, highly educated individuals, and those working or studying in the medical field. This may limit the generalizability of our findings to the broader Egyptian population, such as rural residents, older adults, or people with limited literacy. Future research using more representative sampling designs is recommended to validate and generalize these results. Since the findings are self-reported, they could introduce recall bias, which would influence the validity of the responses. The cross-sectional design of the study makes it challenging to assess causality or to track changes in stroke knowledge over time. The remaining confounding bias may result from unmeasured variables or from variables that are directly or indirectly related to stroke.

## Conclusion

In conclusion, this study showed a good level of stroke knowledge in studied privileged Egyptian subgroups. The observed level of stroke knowledge reflects mainly young, educated, and connected groups, and cannot be extrapolated to the broader Egyptian population. Education, medical background, high income, and familiarity with stroke are the key determinants of good knowledge. Targeted public health interventions should focus on vulnerable populations like older adults, rural residents, and those with lower levels of education to improve their level of knowledge and correct misconceptions and stigma associated with stroke rehabilitation. Future research must tackle the stated limitations and investigate techniques to engage underrepresented groups to obtain generalizable results and ensure a more inclusive stroke education and prevention strategy.

## Data Availability

Data is available upon the reasonable request of the corresponding author.

## Abbreviations

DALYs: Disability-Adjusted Life Years
rt-PA: recombinant tissue plasminogen activator
ACU: Ain Shams University Specialized Hospital
CDC: Centres for Disease Control and Prevention
